# *capB2* Expression Is Associated with *Staphylococcus aureus* Pathogenicity

**DOI:** 10.3389/fmicb.2017.00184

**Published:** 2017-02-10

**Authors:** Dan Li, Yinjuan Guo, Shanshan Wang, Jingnan Lv, Xiuqin Qi, Zengqiang Chen, Lizhong Han, Xueqing Zhang, Liangxing Wang, Fangyou Yu

**Affiliations:** ^1^Department of Laboratory Medicine, the First Affiliated Hospital of Wenzhou Medical UniversityWenzhou, China; ^2^Department of Clinical Microbiology, Ruijin Hospital of Shanghai Jiaotong UniversityShanghai, China; ^3^Department of Respiratory Medicine, the First Affiliated Hospital of Wenzhou Medical UniversityWenzhou, China

**Keywords:** *Staphylococcus aureus*, *capB2*, *sarA*, virulence factor, regulation

## Abstract

CapB2 is recognized as a tyrosine kinase and is likely a vital factor in extracellular polysaccharide synthesis in *Staphylococcus aureus*, but its pathogenicity function and regulatory mechanism remain obscure. Here, we demonstrate that CapB2 enhances bacterial virulence in a murine model. Mice infected with the wild type SA75 strain exhibited significantly larger (*P* < 0.05) skin lesions from days 4 to 7 of infection than those challenged with the *capB2* mutant strain. The effect on virulence was reverted by restoring the *capB2* mutation to the wild type. The related components of the wild type SA75 cell wall in the *capB2* mutant strain (SA75Δ*capB2*) were thinner than wild type SA75 strain and the *capB2* mutant complemented strain (SA75Δ*capB2*-C), which was determined by the transmission electron microscopy. The survival percentages of the wild type strain SA75 and SA75Δ*capB2*-C were significantly higher relative to SA75Δ*capB2*. The results of qRT-PCR studies also indicated that mutations in regulatory gene *sarA* led to a drastic increase in capB2 gene transcription, with a 326-fold increase of growth at 6 h compared with the wild type strain, suggesting that sarA is a major negative regulator of capB2 expression. Taken together, these results demonstrate that the expression of CapB2 promotes *S. aureus* virulence in a mouse model of skin infection, and that *capB2* gene transcription is regulated negatively by SarA.

## Introduction

*Staphylococcus aureus* is a common bacterial pathogen which causes a wide range of human diseases, ranging from relatively benign skin and tissue infections to complicated life threatening diseases such as osteomyelitis, septic arthritis, and bacteremia. *S. aureus* pathogenicity is mediated by bacterial components and secreted virulence factors such as surface-associated adhesins, capsular polysaccharide (CP), and exotoxins. Studies have shown that CP is an important virulence factor in *S. aureus*, and it is believed to enhance microbial virulence (Nilsson et al., 1997; Thakker et al., 1998) in host immune evasion (Nanra et al., 2013).

More than 90% of *S. aureus* strains express CP, and 13 capsular serotypes have been described to date (Arbeit et al., 1984; Karakawa et al., 1985; von Eiff et al., 2007). Polysaccharide capsules corresponding to serotypes 1 and 2 (CP1 and CP2) are associated with heavily encapsulated mucoid strains, but are rarely encountered among clinical isolates (Luong T.T. et al., 2002; Luong et al., 2003). By contrast, CP5 and CP8 are both predominant among clinical infection isolates (Sompolinsky et al., 1985; O’Riordan and Lee, 2004), and are associated with microencapsulated strains exhibiting non-mucoid colony morphology. The *cap5* and *cap8* operons both contain 16 open reading frames, *cap5A1* (*cap8A1*) through *cap5P* (*cap8P*), with the type-specific genes situated in the central region of the operons (*cap5HIJK* or *cap8HIJK*) (Sau et al., 1997a). CapB1, which forms a complex with the C-terminal domain of transmembrane protein CapA1, is probably a regulator of chain length in CP biosynthesis (Sau et al., 1997b), and has been shown to exhibit tyrosine kinase activity (Olivares-Illana et al., 2008). Whole genome sequencing of *S. aureus* has demonstrated that the gene products of *capA2/capB2* (located elsewhere in the genome) are highly similar to those of *capA1/capB1* (Kuroda et al., 2001; Soulat et al., 2006). However, despite CapB1 sharing more than 70% sequence identity with CapB2, no kinase activity could be detected for CapB1 *in vitro*. Of interest, the tyrosine kinase activity of CapB2 is more efficiently activated by the transmembrane stimulatory protein CapA1 than by the corresponding CapA2 activator (Kuroda et al., 2001; Soulat et al., 2006). Once CapB2 has been activated, its tyrosine cluster is phosphorylated (Grangeasse et al., 2012), indicating that CapB2 is probably a vital factor in the synthesis and/or export of extracellular polysaccharides.

The *Staphylococcal* accessory regular (*sar*) locus encodes a DNA binding protein, SarA, which is a key pleiotropic transcriptional regulator for the synthesis of extracellular virulence proteins (Cheung and Zhang, 2002). The *sarA* gene can regulate virulence expression dependently of *agr* (Cheung and Zhang, 2002). And it can also activate certain virulence genes by binding the promoter of the target gene directly (Cheung and Zhang, 2002).

In the present study, we analyzed the role of *capB2* in the virulence of *S. aureus* by constructed an allelic replacement mutant of *S. aureus* 75. Our results show that a *capB2* mutant of *S. aureus* is less virulence in the mice model of skin infection. In addition, we investigated the *capB2* expression regulated by SarA.

## Materials and Methods

### Bacterial Strains, Plasmids, and Culture Conditions

The bacterial strains and plasmids used in this study are described in **Table 1**. *S. aureus* strain SA75 was isolated from a patient with a purulent skin infection at the First Affiliated Hospital in Wenzhou. Unless otherwise stated, *Escherichia coli* strains were cultured in Luria-Bertani medium, and *S. aureus* strains were grown in tryptic soy broth (TSB) medium, at 37°C with shaking at 200 rpm. The culture media were supplemented with appropriate antibiotics when required (ampicillin at 100 mg/l, chloramphenicol at 10 mg/l and anhydrotetracycline at 50 ng/ml).

**Table 1:** Bacterial strains and plasmids used in this study.

Strains and plasmixds	Description	Source
**Strains**		
*S. aureus*		
SA75	Wild type, CP8-positive, clinical MRSA strain isolated from an abscess	Laboratory stock
SA75Δ*sarA*	Isogenic *sarA* deletion mutant in SA75	Laboratory stock
SA75Δ*capB2*	Isogenic *capB2* deletion mutant in SA75	This study
SA75Δ*sarA*-C	*sarA* mutant complemented with pRB*sarA*	This study
SA75Δ*capB2*-C	*capB2* mutant complemented with pRB*capB2*	This study
*E. coli*		
DH5α	Clone host strain	Laboratory stock
DC10B	dam+Δ*dcm*^-^Δ*hsdRMS* endA1 recA1; clone host strain	Laboratory stock
**Plasmids**		
pKOR1	Shuttle cloning vector, temp sensitive (Cm^r^Amp^r^)^a^	Laboratory stock
pK*capB2*	pKOR1 containing fragments 1500-bp upstream and 1500-bp downstream of *capB2* gene, for *capB2* mutagenesis (Cm^r^Amp^r^)	This study
pRB473	Shuttle cloning vector (Cm^r^)	Laboratory stock
pRB*sarA*	pRB473 with *sarA* and its promoter (Cm^r^)	This study
pRB*capB2*	pRB473 with *capB2* and its promoter (Cm^r^)	This study


*^*a*^Cm^*r*^Amp^*r*^, chloramphenical and ampicillin resistance.*

### DNA Manipulations

Genomic DNA extraction was performed as described previously (Yu et al., 2014). Plasmid DNA extracted from *E. coli* strains was purified according to the manufacturer’s instruction (Axygen, Union City, CA, USA). To extract plasmid DNA from *S. aureus* strains, 20 μl lysostaphin (2 mg/ml) was used with suspension buffer S1 to re-suspend the collected cells from 10 ml overnight culture, incubated at 37°C for 1 h, and then processed following the manufacturer’s instruction (TIANGEN, Beijing, China).

### Construction of *S. aureus capB2* Mutant Strain by Allelic Replacement

In order to generate a *capB2* mutant from SA75, genomic DNA was extracted from *S. aureus* SA75 and the two sets of primers listed in **Table 2** were used to amplify DNA fragments 1.5-kb upstream and downstream of *capB2* from SA75 chromosomal DNA. The amplified products were digested with *Hin*dIII and then ligated with T4 DNA ligase to yield a 693 bp deletion fragment (called Δ*capB2*). The primers UP-F-attB2 and Dn-R-attB1 were used to amplify Δ*capB2* fragment, yielding a PCR product with *attB* sites at the 5′ and 3′ ends. The resulting fragments were recombined with temperature-sensitive shuttle plasmid pKOR1 to generate recombinant plasmid pK*capB2*.

**Table 2:** Primers used in this study.

Primers	Oligonucleotide (5′→3′)^a^
*capB2*-up-F- BamHI	CGggatccGCACGCTCGGTGTTGTAAAG
*capB2*-up-R- HindIII	CCCaagcttCTTCTTCGTGTATTCGTCAT
*capB2*-down-F- HindIII	CCCaagcttGCATATTATGGGACTGATGA
*capB2*-down-R- XhoI	CCGctcgagTTGATCTAAGTCCTGCATGA
UP-F-attB2	GGGGACCACTTTGTACAAGAAAGCTGGGTGCACGCTCGGTGTTGTAAAG
Dn-R-attB1	GGGGACAAGTTTGTACAAAAAAGCAGGCTTTGATCTAAGTCCTGCATGA
SW-cp-F	ATAGTTTAGTAAGCGACCTG
XW-cp-R	TACAGGAAATCCGCATAAAT
Cp-BD-F	ATGACGAATACACGAAGAAG
Cp-BD-R	TCATGATTCATCAGTCCCAT
PstI-*sarA*-F	AAAActgcagTAAACCAAATGCTAACCCA
BamHI-*sarA*-R	CGCggatccAGTGCCATTAGTGCAAAAC
PstI-*cap*-19F	AAAActgcagATTTTTGTTACTAGTTTGT
BamHI-*cap*-19R	CGggatccTCATGATTCATCAGTCCCA
*cap5K*-F	GTCAAAGATTATGTGATGCTACTGAG
*cap5K*-R	ACTTCGAATATAAACTTGAATCAATGTTATACAG
*cap8I*-F	GCCTTATGTTAGGTGATAAACC
*cap8I*-R	GGAAAAACACTATCATAGCAGG
*gyrB*-F	ACATTACAGCAGCGTATTAG
*gyrB*-R	CTCATAGTGATAGGAGTCTTCT
*cap*-XF	ACGAATACACGAAGAAGTA
*cap*-XR	CCTCTGAAGTGATTACAATG
*sarA*-qtF	GTTATCAATGGTCACTTATGC
*sarA*-qtR	CTTGTGGTTGTTTGTAGTTT


*^a^Restriction sites are indicated by lowercase letters; underlined sequences denote recombination sites.*

The resultant plasmid containing the Δ*capB2* gene deletion was transferred first to *E. coli* DH5α and then to *E. coli* DC10B, and subsequently electroporated into strain SA75. The allelic exchange procedure was performed as described previously (Bae and Schneewind, 2006). Putative transformants were screened by PCR with LATaq DNA polymerase, using primer pairs SW-cp-F/XW-cp-R and Cp-BD-F/Cp-BD-R. Deletion of the desired gene was verified by PCR, quantitative reverse transcription-PCR (qRT-PCR), and sequencing.

### Construction of Complemented Strains

For complementation of the *sarA* gene and *capB2* gene, the deleted genes and their own putative promoters region were amplified by PCR with Iproof High-Fidelity DNA polymerase using primers PstI-*sarA*-F and BamHI-*sarA*-R for *sarA*, and primers PstI-*cap*-19F and BamHI-*cap*-19R for *capB2*. The PCR products were digested with *Bam*HI and *Pst*I, and the fragments were ligated into shuttle plasmid pRB473 to generate plasmid pRB*sarA* and pRB*capB2*. The resultant plasmids were transferred into *E. coli* DH5α and DC10B successively, and finally electroporated into mutant strains SA75Δ*sarA* and SA75Δ*capB2*. Successful uptake of the complementation plasmid was confirmed by restriction mapping, PCR and sequencing of PCR fragments. The presence of *sarA* transcription or *capB2* transcription within the transformants was verified by qRT-PCR.

### Capsular Genotype Detection

Capsular genotype was detected by PCR as described previously (Verdier et al., 2007). Genomic DNA from strain SA75 was used as template, with primers *cap5k*-F and *cap5k*-R for detection of capsular type 5, and primers *cap8I*-F and *cap8I*-R for detection of capsular type 8. PCR product sizes were 361 and 173 bp for capsular types 5 and 8, respectively, and were confirmed by sequencing.

### Growth Curve

*Staphylococcus aureus* wild type strain SA75 and its derivative strains were incubated overnight in 5 ml TSB at 37°C with shaking at 200 rpm. The overnight cultures were diluted in 30 ml TSB to obtain the same starting optical density (OD) at 562 nm. The growth of each strain was monitored by Microplate Manager 6 (Bio-Rad, USA) software at 1 h intervals for a total of 12 h.

### *S. aureus* Survival Analyze in Human Blood

Blood samples from healthy donors were collected in 10 mg/ml heparin anticoagulation tubes. The ability of *S. aureus* SA75 and SA75Δ*capB2* to survive in human blood was determined under post-logarithmic growth phase. The strains were analyzed after washed twice and suspended to an OD of 0.6 at 562 nm. Then, 1 ml of heparinized human blood was inoculated with 100 μl aliquot of these suspensions and was incubated at 37°C. After 7 h, bacteria cells were appropriate diluted to detect the endpoint numbers of CFU. The survival rate of the bacteria cells was determined by comparing to the initial inoculum.

### Mouse Model of Skin Infection Assay

Animal studies were approved by the Institutional Animal Care and Use Committee at Wenzhou Medical University. We confirm that all animal care and methods were performed in accordance with the guidelines and regulations approved by the Administration of Affairs Concerning Experimental Animals in China. The skin abscess model was performed as described previously (Li et al., 2012). Female, 4- to 6-week old, BALB/C-nu mice were used in the study. The mice were housed for 7 days before inoculation with food and water *ad libitum. S. aureus* cultures were grown to post-exponential phase, washed with sterile phosphate-buffered saline (PBS), and then suspended in PBS to achieve a concentration of 1.0 × 10^5^ CFU/100 μl. The bacterial numbers were confirmed by plate counts. After the mice were anesthetized with diethyl ether, each mouse was injected subcutaneously with 100 μl of either *S. aureus* suspension or PBS. Skin lesions developing on test animals were observed daily, and the abscess area calculated using the formula length (L) × width (W). The values of L and W for each infected mouse were determined by caliper. Dermonecrosis was observed and recorded for 10 days, following which all animals were euthanized.

### Electron Microscopy Study on Abscess Tissue

Overnight cultures of *S. aureus* SA75, *capB2* mutant and the complemented strains were harvested and washed twice with sterile PBS, and then suspended to achieve a concentration of 1.0 × 10^8^ CFU/100 μl. Mouse model of subcutaneous abscess was performed as above. The abscess tissue was obtained at the fourth day of infection. Samples were fixed and processed further for transmission electron microscopy.

### RNA Extraction, cDNA Synthesis, and Quantitative Reverse Transcription-PCR (qRT-PCR)

The quantitative Real-Time PCR was performed according to the MIQE guidelines. For RNA isolation, *S. aureus* strains were diluted 1:100 in TSB from overnight cultures and grown to post-exponential phase at 37°C with shaking at 200 rpm. Total RNA was isolated and purified using a PureLink RNA Mini Kit (Invitrogen, Carlsbad, CA, USA); 2 μg purified RNA was then used to obtain cDNA using a PrimeScript RT reagent kit (TaKaRa, Japan) following the manufacturer’s instructions. The resultant cDNA was amplified using a SsoFas EvaGreen Supermix kit (Bio-Rad, USA) with the Bio-Rad CFX96 Manager software. Oligonucleotide primers used are listed in **Table 2**. Wild type strain SA75 was used as a control (relative expression = 1), and *gyrB* was used as a reference gene to investigate genes of interest. RNA transcript levels were calculated by the method of *delta delta* Ct (ΔΔCt) (Montgomery et al., 2012). Data analysis was carried out using Bio-Rad CFX software. Each reaction was performed in triplicate.

### Statistical Analysis

Skin abscesses in the mouse infection model were compared with one-way analysis of variance (ANOVA) using SPSS Statistics 17.0. Results were considered statistically significant if *P-*values were < 0.05. Relative expression levels resulting from qRT-PCR were analyzed using GraphPad Prism 5.0 software.

## Results

### Capsular Genotype and Growth Curve Analysis

In order to rule out the potential influence of the *capB2* deletion on bacterial growth rate, the growth curves of strains SA75, SA75Δ*capB2*, and SA75Δ*capB2*-C were monitored hourly. The growth curves of the three strains were measured in TSB medium at the same starting OD of 562 nm, with TSB medium used as a blank control. There were no substantial differences in growth curves among all three strains. The *capB2* gene was expressed at the indicated time point (3, 6, 9, 12 h) and assessed in strain SA75, with expression shown to be highest at the 6 h time point.

### Deletion of *capB2* in *S. aureus*

**FIGURE 1:**
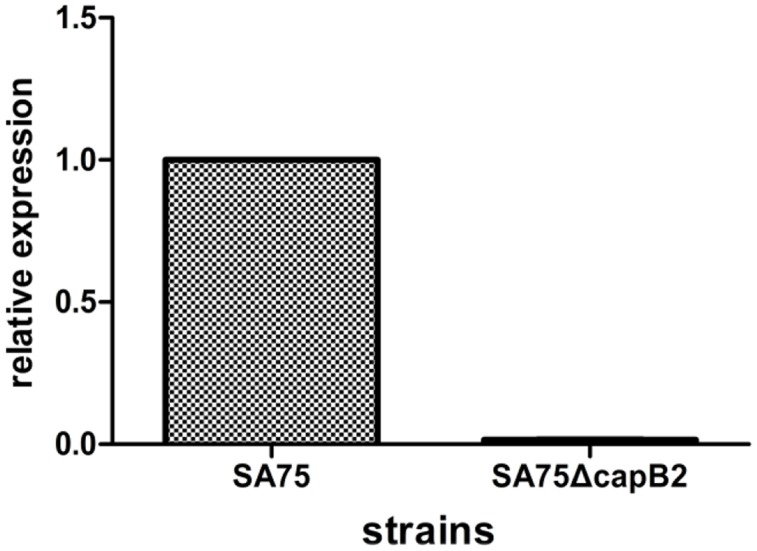
**Deletion of *capB2* in *S. aureus*.** Gene *gyrB* was used as the reference gene; the expression of *capB2* in strain SA75 was regarded as 1.

The *capB2* deletion was introduced by allelic replacement into *S. aureus* strain SA75, yielding the mutant strain SA75Δ*capB2*. High-efficiency cloning strain *E. coli* DC10B was used as an intermediate host instead of *S. aureus* RN4220 in the study in order to improve the transformation efficiency. To confirm the deletion of *capB2*,SA75Δ*capB2* was amplified with primers SW-cp-F and XW-cp-R using LA Taq DNA polymerase. The band size in strain SA75Δ*capB2* reflected a 693 bp deletion compared to the wild type strain. The relative expression of *capB2* in strain SA75Δ*capB2* was dramatically decreased relative to that of strain SA75 (**Figure 1**).

### Absence of *capB2* Decreased *S. aureus* Viability in Human Blood

**FIGURE 2:**
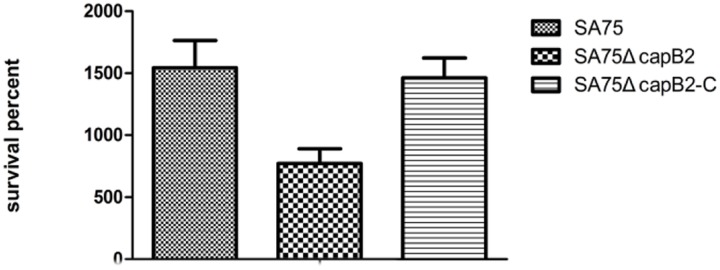
**The suvival rate of *S. aureus* derivatives in human blood.** The number of CFU was detected at 0 and 7 h to calculate the rates of survival for *S. aureus* strains SA75, SA75Δ*capB2*, and SA75Δ*capB2*-C exposed to human blood.

To determine the contribution of *capB2* in the pathogenicity of *S. aureu*s, we compared the relative viability of *S. aureus* SA75 and SA75Δ*capB2* in human blood. After 7 h incubation, the survival percentages of the wild type strain SA75 and the complemented strain SA75Δ*capB2*-C were significantly higher relative to SA75Δ*capB2* (**Figure 2**).

### Deletion of *capB2* Decreases Virulence of *S. aureus* SA75 in a Skin Infection Model

**FIGURE 3:**
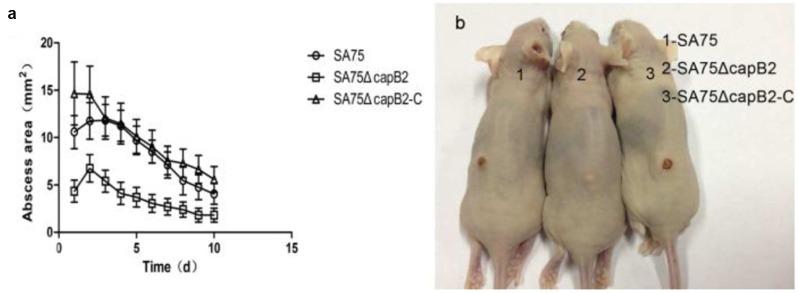
**Results of mouse skin infection model experiments.** **(a)** Comparison of abscess size (area) of BALB/C-nu mice infected with wild type (SA75), isogenic capB2 mutant (SA75ΔcapB2), and capB2-complemented (SA75ΔcapB2-C) strains strains; **(b)** Skin lesions resulting from *S. aureus* infection (picture taken at day 10 of infection). Differences in the size/area of skin abscesses were compared by one-way analysis of variance (ANOVA) using SPSS Statistics 17.0.

To determine the role of *capB2* in the pathogenesis of *S. aureus*, 4-to-6 week old BALB/C-nu mice were subcutaneously injected with 1.0 × 10^5^ CFU of strains SA75, SA75Δ*capB2*, or SA75Δ*capB2*-C. As shown in **Figure 3b**, the resulting abscess sizes in mice inoculated with the *capB2* mutant strain were smaller than the lesion sizes among animals infected with SA75 or the complemented strain SA75Δ*capB2*-C. The lesions remained until the end point of the experiment (**Figure 3a**). The overall rates of skin lesions were similar between the wild type and the complemented strain.

As shown in **Figure 3a**, animals challenged with 1.0 × 10^5^ CFU of SA75 had significantly larger (*P* < 0.05) skin lesions from the fourth day to the seventh day of infection than animals challenged with an equivalent amount of SA75Δ*capB* Likewise, significant differences between the SA75Δ*capB* and the SA75Δ*capB*-C strains were observed from the second day to the ninth day of infection. These data demonstrate that the presence of *capB2* in *S. aureus* promotes bacterial virulence.

### Electron Microscopy Study

**FIGURE 4:**
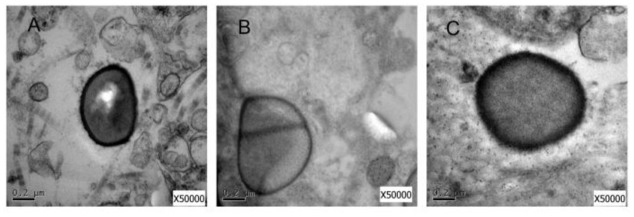
**Electron microscopy study.** **(A)** Transmission electron micrograph of cell walls of *S. aureus* SA75 **(B)** the *capB2* mutant strain **(C)** and the complement strain.

To analyze the influence of the deletion of *capB2* gene in *S. aureus* SA75, we used the transmission electron microscopy to detect the difference between *S. aureus* SA75, SA75Δ*capB* and SA75Δ*capB*-C strains. As shown in **Figure 4**, the related components of *S. aureus* SA75 cell wall in SA75Δ*capB* was thinner than SA75 and SA75Δ*capB*-C.

### Effect of *sarA* on *capB2* Expression

**FIGURE 5:**
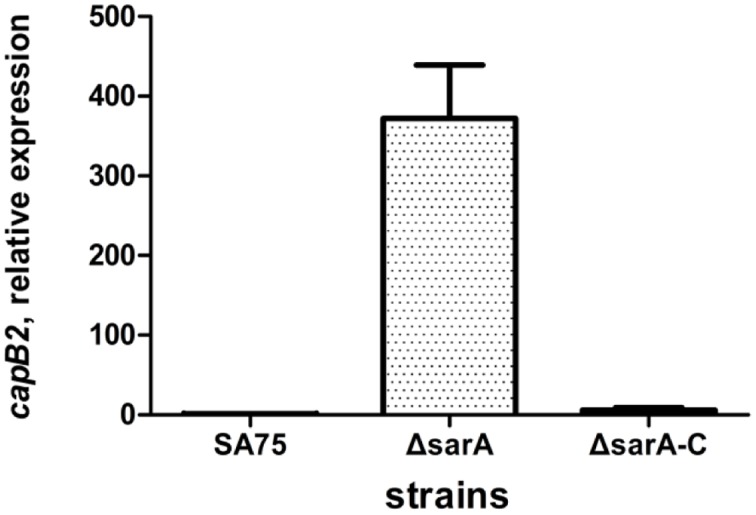
**Effect of *sarA* on expression of *capB2*.** Relative transcription of the *capB2* gene was detected by qRT-PCR. Reference gene *gyrB* was used as the relative expression gene; the expression of *capB2* in strain SA75 was regarded as 1.

In our previous work, we constructed a *sarA* mutant strain. To eliminate the possibility of a second mutant, we constructed a *sarA*-complemented strain containing a plasmid bearing the *sarA* gene. Since the transcriptional expression of *capB2* was highest at the 6 h time point, we selected that time point to assess expression differences among the three strains. The effect of *sarA* on expression of *capB2* was assessed by qRT-PCR. As shown in **Figure 5**, deletion of *sarA* had a dramatic effect on *capB2* expression, with a 326-fold increase at 6 h of growth compared with that of the wild type strain. The difference in *capB2* expression was thus reversed by complementation of *sarA*. These results suggest that *sarA* represses the expression of *capB2*.

## Discussion

Capsular polysaccharide produced by *S. aureus* has been acknowledged as an important virulence factor for bacterial infection. Methods for *S. aureus* capsule serotyping include enzyme-linked immune sorbent assay (ELISA) inhibition, colony immune blotting, and double immune diffusion assay (the gold standard). However, all of them are based on the availability of specialized antibodies, and are not suitable for rapid capsule serotyping. Grunert et al. (2013) have developed a high-throughput method to identify capsular serotypes 5, 8 and NT (non-typable). DNA sequence analysis of *S. aureus* has determined that the *cap5HIJK* and *cap8HIJK* genes are the primary capsular type-specific genes. This information allowed us to identify the capsular genotype of SA75 using type-specific oligonucleotide primers (Verdier et al., 2007) designed against the *cap5K* and *cap8I* genes, with the results demonstrating that SA75 belongs to capsular type CP8.

Since the skin infection model was shown to be more sensitive, the role of CapB2 in *S. aureus* pathogenicity was evaluated using the skin infection model. Notably, the *capB2* deletion decreased virulence in the mouse model of *S. aureus* skin infection, but full virulence was restored by transformation with the complementation plasmid containing the *capB2* gene. Neither the *capB2* mutant strain nor the complementation strain showed any difference in bacterial growth compared with the wild type strain when grown in TSB medium. Of important, the mutant of *capB2* in *S. aureus* strain resulted in difference in the related components of cell wall. The role of CP in the pathogenesis of *S. aureus* infection has been reported by a number of studies (Baddour et al., 1992; Luong and Lee, 2002; O’Riordan and Lee, 2004; Nanra et al., 2013), which found that expression of CP5 or CP8 can enhance bacterial virulence both *in vitro* and *in vivo*. Taken together, we conclude that the decrease in virulence of the *capB2* mutant strain is likely due to decreased synthesis of CP rather than the effect on overall growth.

Similar to other virulence proteins, CP and CapB2 are also regulated by a complex regulatory network. Luong T. et al. (2002) investigated the regulation of *S. aureus* CP8 expression by the global regulators *agr* and *sarA*, and found that the CP8 expression was positively regulated by *agr* and *sarA*, with *agr* being the major activator. However, our results demonstrate that *capB2* expression was dramatically repressed by *sarA*, suggesting that SarA is a negative regulator of *capB2* expression. The difference in regulation may be due to the complex regulatory network present in *S. aureus*, with the *sarA* interface within the regulatory network controlling the expression of virulence-associated proteins (Arvidson and Tegmark, 2001). In addition, the expression of CP is also influenced by several regulatory proteins. The regulation of CP by the two-component system KdpDE has been reported by Zhao et al. (2010), who found that CP expression was reduced in the *kdpDE* mutant strain, and that positive regulation occurred *via* the phosphorylation of KdpE. Furthermore, Lei and Lee (2015) found that RbsR (a Lacl family of repressor) positively regulates *cap* gene expression by directly binding to the *cap* operon promoter region, as evidenced by electrophoretic mobility shift assays (EMSA). In brief, the complexity of virulence regulatory networks in *S. aureus* is such that a single virulence protein may be controlled by a remarkable number of regulators.

In summary, CapB2 is a probable virulence gene in *S. aureus* infection, with deletion of *capB2* resulting in decreased virulence in a mouse model of skin infection. SarA, on the other hand, is a negative regulator of *capB2* expression, but further in-depth studies are required to determine if it directly regulates *capB2*.

## Ethics Statement

This study was reviewed by the Ethics Committee of the first Affiliated Hospital of Wenzhou Medical University.

## Author Contributions

DL performed the experiments, wrote the main manuscript text and analyzed the data. YG, SW, ZW, JL, and XQ helped to conduct and perform the experiments. ZC, LH, and XZ contributed to preparing the reagents/materials and supervised the project. LW and FY conceived the experiments and helped with the discussion of results. All authors reviewed the manuscript.

## Conflict of Interest Statement

The authors declare that the research was conducted in the absence of any commercial or financial relationships that could be construed as a potential conflict of interest.
